# Ocular Syphilis With Unilateral Optic Papillitis and Outer Retinopathy Complicated by Diabetic Retinopathy

**DOI:** 10.7759/cureus.72534

**Published:** 2024-10-28

**Authors:** Kyosuke Nonaka, Yohei Takahashi, Taiji Hasegawa, Tomohiro Iida

**Affiliations:** 1 Ophthalmology, Tokyo Women's Medical University, Tokyo, JPN; 2 Ophthalmology, Tokyo Women’s Medical University, Tokyo, JPN

**Keywords:** neurosyphilis, ocular syphilis, optic neuritis, papillitis, syphilis

## Abstract

Syphilis has been increasing in adult infections in recent years, and ocular syphilis includes not only uveitis but also a variety of optic nerve and retinal lesions. We report a case of syphilis that caused unilateral optic papillitis and outer retinopathy complicated by diabetic retinopathy and improved with antibiotic treatment. The patient was a 61-year-old woman. During follow-up after vitreous surgery for proliferative diabetic retinopathy, her left visual acuity had decreased, fundus examination revealed redness and swelling of the left optic disc, and a visual field test revealed a central scotoma. Fluorescein angiography (FA) revealed leakage from the optic disc, and magnetic resonance imaging (MRI) revealed high signal intensity and contrast enhancement in the left optic nerve. No signs of uveitis, and the patient was diagnosed with optic neuritis and one course of steroid pulse therapy was performed. Due to improvement of the left optic disc swelling and MRI contrast enhancement, the oral steroids were gradually tapered and discontinued after about one month due to improvement in left optic disc swelling and MRI contrast enhancement. Meanwhile, blood tests during steroid administration revealed quantitative positive syphilis lipid antibodies and treponemal antibodies, and optical coherence tomography (OCT) revealed disruption in the left ellipsoid zone. Early acquired syphilis was diagnosed, and oral penicillin antibiotics were started. Five weeks after starting antibiotics, the outer retinal disruption showed signs of improvement. Ocular syphilis presents with characteristic retinal disorders, but in cases where the onset is optic disc edema, early diagnosis is difficult, and careful differentiation is required.

## Introduction

Syphilis is a sexually transmitted infection caused by the bacterium *Treponema pallidum*. It progresses through four stages: primary, secondary, latent, and tertiary syphilis. The infection can affect multiple organs, including the nervous system, cardiovascular system, and eyes, leading to a variety of clinical manifestations [1]. Contrary to congenital syphilis, which is transmitted through the placenta, acquired syphilis is a sexually transmitted disease, and cases of co-infection with human immunodeficiency virus (HIV) have been reported [2,3], making it a clinical problem. Advances in anti-syphilis therapy with penicillin antibiotics have significantly reduced the incidence of acquired syphilis, especially in developed countries. Traditionally, most cases were caused by homosexual intercourse between males, but in recent years, the number of acquired infected cases of syphilis has been increasing, especially among adult females [4].

Ocular syphilis refers to an infection of the eyes caused by *Treponema pallidum*, which can occur in various stages of syphilis. It presents with a wide range of symptoms, including uveitis (inflammation of the middle layer of the eye), keratitis (corneal inflammation), optic neuritis (inflammation of the optic nerve), and retinal disorders [5], which may lead to vision loss if untreated. The most common symptoms are uveitis, chorioretinitis, and retinal vasculitis, but optic nerve diseases can also occur, and there have been occasional reports of cases of optic disc edema [3,6-8]. Optic papillitis, or inflammation of the optic disc, is typically seen bilaterally in cases of ocular syphilis. However, unilateral presentations are rare, making this case notable and underscoring the complexity of diagnosing and managing such cases. Neurosyphilis can cause irreversible optic nerve atrophy, so early detection and initiation of treatment are important [9].

On the other hand, acute syphilitic posterior placoid chorioretinitis (ASPPC), proposed by Gass et al. in 1990, is known as a specific fundus finding due to syphilis, which is a yellowish finding of the outer retina seen in the macula [10]. Optical coherence tomography (OCT) findings show characteristics of outer retinopathy, such as disruption of the ellipsoid zone (EZ)/interdigitation zone (IZ), loss of the external limiting membrane (ELM), nodular thickening and hyperreflectivity of the retinal pigment epithelium (RPE), and punctate hyperreflectivity of the choroid [11,12].

Ocular syphilis has few characteristic findings and is often misdiagnosed as other ocular diseases or a definitive diagnosis is delayed. In particular, there have been reports of cases in which it was difficult to distinguish from other retinal diseases, such as acute zonal occult outer retinopathy (AZOOR) and Behçet's disease [13,14]. Diabetic retinopathy is a complication of diabetes that affects the eyes, leading to damage of the blood vessels in the retina. This condition can result in vision impairment or blindness if not properly managed. When present alongside infections such as syphilis, it complicates the diagnosis and treatment of ocular conditions. In this case, we report a patient with syphilis who presented with unilateral optic papillitis and subsequent outer retinopathy during the course of diabetic retinopathy, and the findings improved with antibiotic treatment.

## Case presentation

The patient, a 61-year-old woman with poorly controlled diabetes, presented with decreased visual acuity in the left eye. She had a history of vitreous surgery for vitreous hemorrhage and anti-vascular endothelial growth factor (VEGF) intravitreal injection for macular edema; her macular edema due to diabetic retinopathy had resolved, but her visual acuity in both eyes remained approximately 20/32. Upon examination at the time of the visit, the best corrected visual acuity (BCVA) was 20/40 in the right eye and 20/50 in the left eye. The pupillary light reflex was slightly sluggish, with no difference between the left and right eyes, and the relative afferent pupillary defect (RAPD) was negative. The critical flicker frequency (CFF) had decreased to 16 Hz, and a central scotoma was noted during visual field testing of the Goldmann perimeter (GP) in the left eye.

**Figure 1:**
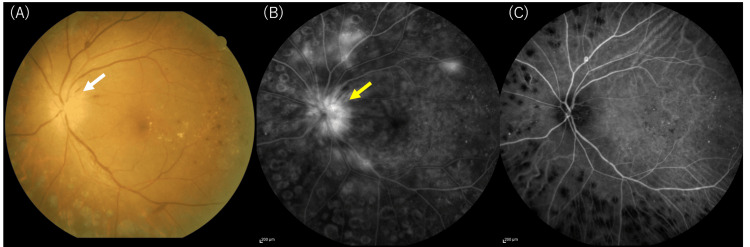
Fundus photo and angiography images of the left eye before treatment. This fundus photo shows optic disc swelling (white arrow) in the left eye with scattered retinal hemorrhages and white deposits due to diabetic retinopathy. Fluorescein angiography (FA) reveals optic disc leakage (yellow arrow) (B), and indocyanine green angiography (ICGA) shows no delayed choroidal filling, ruling out ischemic optic neuropathy (C).

**Figure 2:**
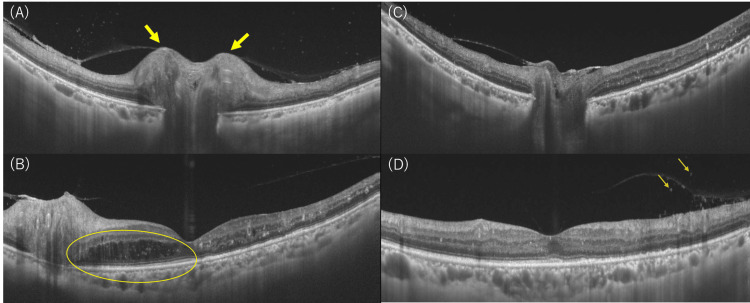
Optical coherence tomography (OCT) images of the left eye before treatment and after steroid pulse. Optical coherence tomography (OCT) reveals swelling around the optic disc (yellow arrows) (A) and retinal edema (yellow circle) (B) between the optic disc and macula before treatment. After steroid pulse therapy, the optic disc swelling subsides (C), and inflammatory cells appear in the vitreous (arrows in (D)). The visual acuity in the left eye before treatment was 20/50, whereas the visual acuity after the steroid pulse was 20/40.

Fundus photography of the left eye revealed new optic disc swelling in addition to scattered retinal hemorrhage and white spot deposits, all associated with diabetic retinopathy (Figure 1A). No signs of uveitis were observed. No optic disc swelling was observed in the right eye, and differential diagnoses for the unilateral optic disc swelling included optic neuritis (papillitis), peri-optic neuritis, anterior ischemic optic neuropathy, and diabetic optic neuropathy. Fluorescein angiography (FA) showed fluorescence leakage from the left optic disc (Figure 1B). Indocyanine green angiography (ICGA) showed no obvious delayed choroidal filling around the left optic disc, and no findings suggestive of anterior ischemic optic neuropathy were observed (Figure 1C). At this time, no retinal placoid lesions or corresponding abnormal hyperfluorescent or hypofluorescent spots on FA and ICGA were noted. OCT findings showed swelling around the optic disc in the left eye, and retinal edema between the optic disc and the macula (Figures 2A, 2B). At this time, OCT findings of the outer retina, EZ, ELM, and RPE, around the macula did not reveal any abnormalities. The retinal edema (Figure 2B) was considered to be due to optic disc swelling (Figure 2A) and not an effect of diabetic retinopathy. In the right eye, no optic disc swelling or macular edema was observed.

**Figure 3:**
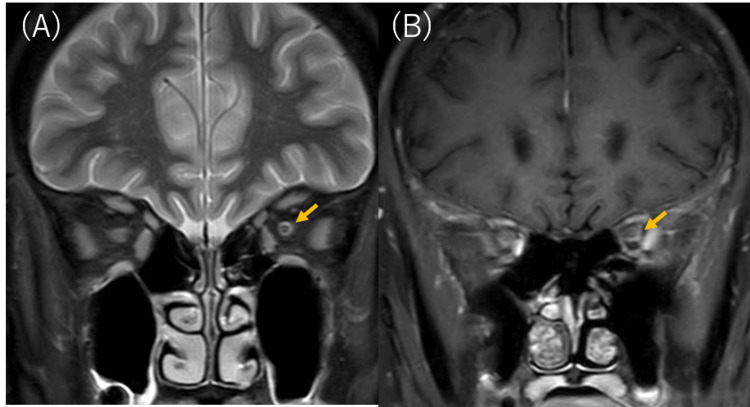
Orbital magnetic resonance imaging (MRI) images before treatment. (A) On T1-weighted with fat suppression (short tau inversion recovery: STIR), the mild high signal intensity of the left optic nerve (arrow). (B) On T1-weighted gadolinium-enhanced images, slight suggestion of contrast enhancement around the left optic nerve (arrow).

Orbital magnetic resonance imaging (MRI) was performed to diagnose optic neuritis. On T1-weighted with fat suppression (short tau inversion recovery: STIR) and gadolinium-enhanced images, mild hyperintensity and slight suggestion of contrast enhancement were observed around the left optic nerve (Figure 3). No high signal intensity or contrast enhancement was evident in the right optic nerve. The contrast enhancement in the left optic nerve was most suggestive of acute optic neuritis, and anterior ischemic optic neuropathy without contrast enhancement was excluded. Multiple sclerosis and other conditions were also excluded because there were no findings such as intracerebral plaques. Unilateral left optic neuritis was mostly suspected, and the patient was admitted to the hospital and administered one course of methylprednisolone at 1,000 mg for three days as a steroid pulse. Laboratory data at the start of steroid treatment showed a worsening of diabetes with HbA1c of 12.8%, and a request was made for blood sugar control management by an internal medicine department.

After steroid pulse treatment, visual acuity remained unchanged at 20/40 in both eyes. As autoantibodies, including aquaporin-4 (AQP4) antibodies, were confirmed to be negative, the patient was diagnosed with suspected idiopathic optic neuritis and was discharged after starting follow-up oral steroids at 30 mg/day. To prevent the worsening of concomitant diabetes, the steroid dose was tapered relatively rapidly by 10 mg/day every week, and within one month of starting oral administration, it was tapered to 5 mg/day. During the course of the treatment, OCT showed improvement in the left optic disc swelling (Figure 2C), while a small number of inflammatory cells appeared in the vitreous (Figure 2D). Re-examination of the orbital MRI showed no high signal intensity or contrast enhancement in either optic nerve. We determined that the improvement of the left optic disc swelling on OCT and the disappearance of contrast enhancement on MRI correlated with the clinical resolution of the left optic neuritis due to the effect of steroids, and oral steroids were discontinued.

On the other hand, qualitative syphilis serological tests, both the rapid plasma reagin (RPR) test to detect lipid antibodies and the *Treponema pallidum* hemagglutination (TPHA) test to detect treponemal antibodies, were positive, and subsequent quantitative retests confirmed high values (RPR: 131.5 R.U. and TPHA: 338.0 COI). There was no increase in the inflammatory reaction, and other infectious diseases, including HIV, were negative. As a result of referral to the urology department for concurrent symptoms of frequent urination, it was found that the patient had a history of sexual contact just before the onset of optic papillitis, and the patient was diagnosed with early acquired syphilis and started on the penicillin antibiotic amoxicillin, 1,500 mg/day.

**Figure 4:**
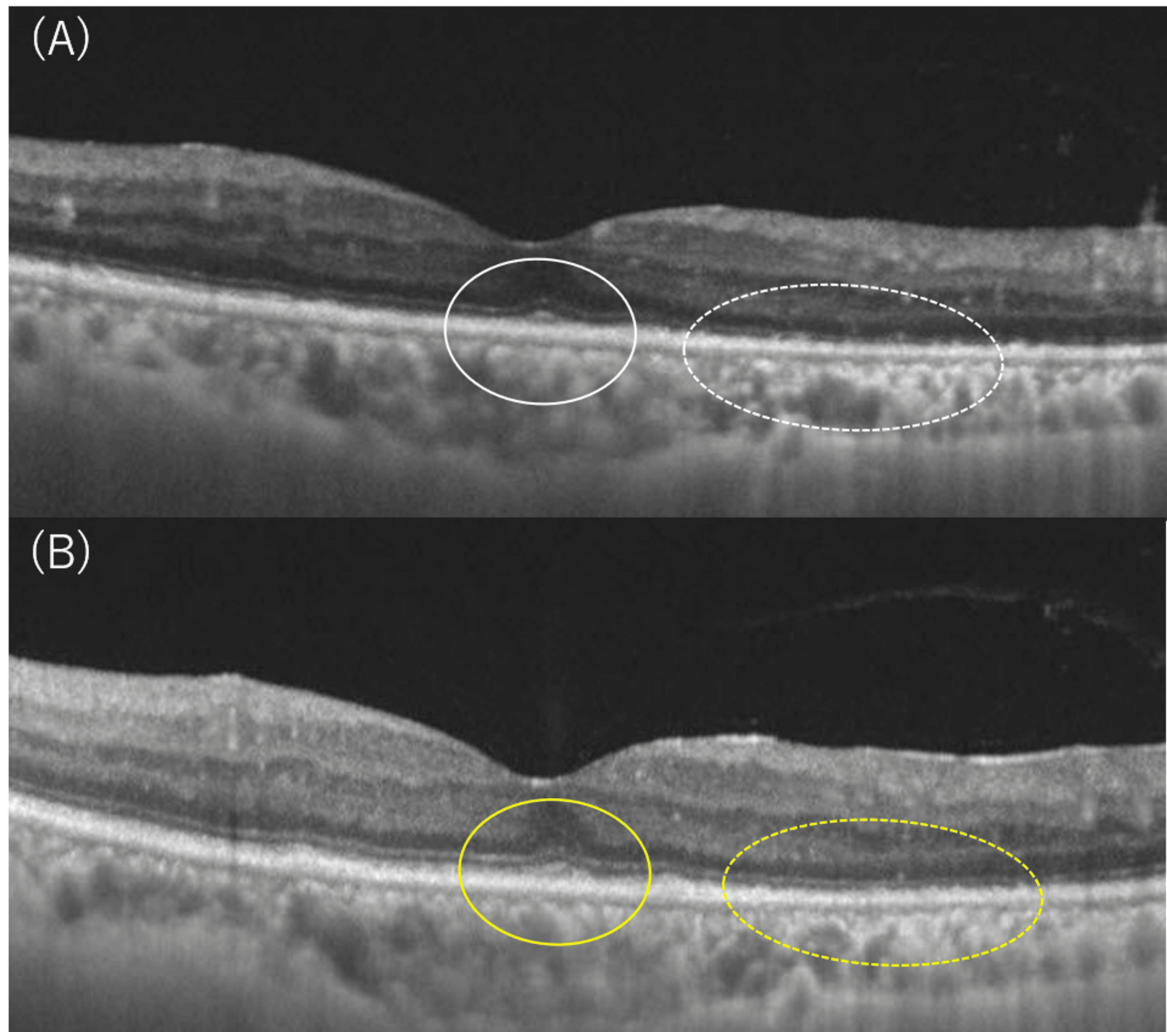
Changes in OCT findings in the left eye while continuing antibiotics after a positive diagnosis of syphilis. (A) OCT of the left eye showed destruction and loss of the macular EZ (circle) and inferior ELM (dotted circle), with visual acuity decreasing to 20/80. (B) After five weeks of continued antibiotic treatment, destruction and loss of the EZ (circle) and ELM (dotted circle) gradually improved, and visual acuity improved to 20/50. OCT: optical coherence tomography, EZ: ellipsoid zone, ELM: external limiting membrane.

**Figure 5:**
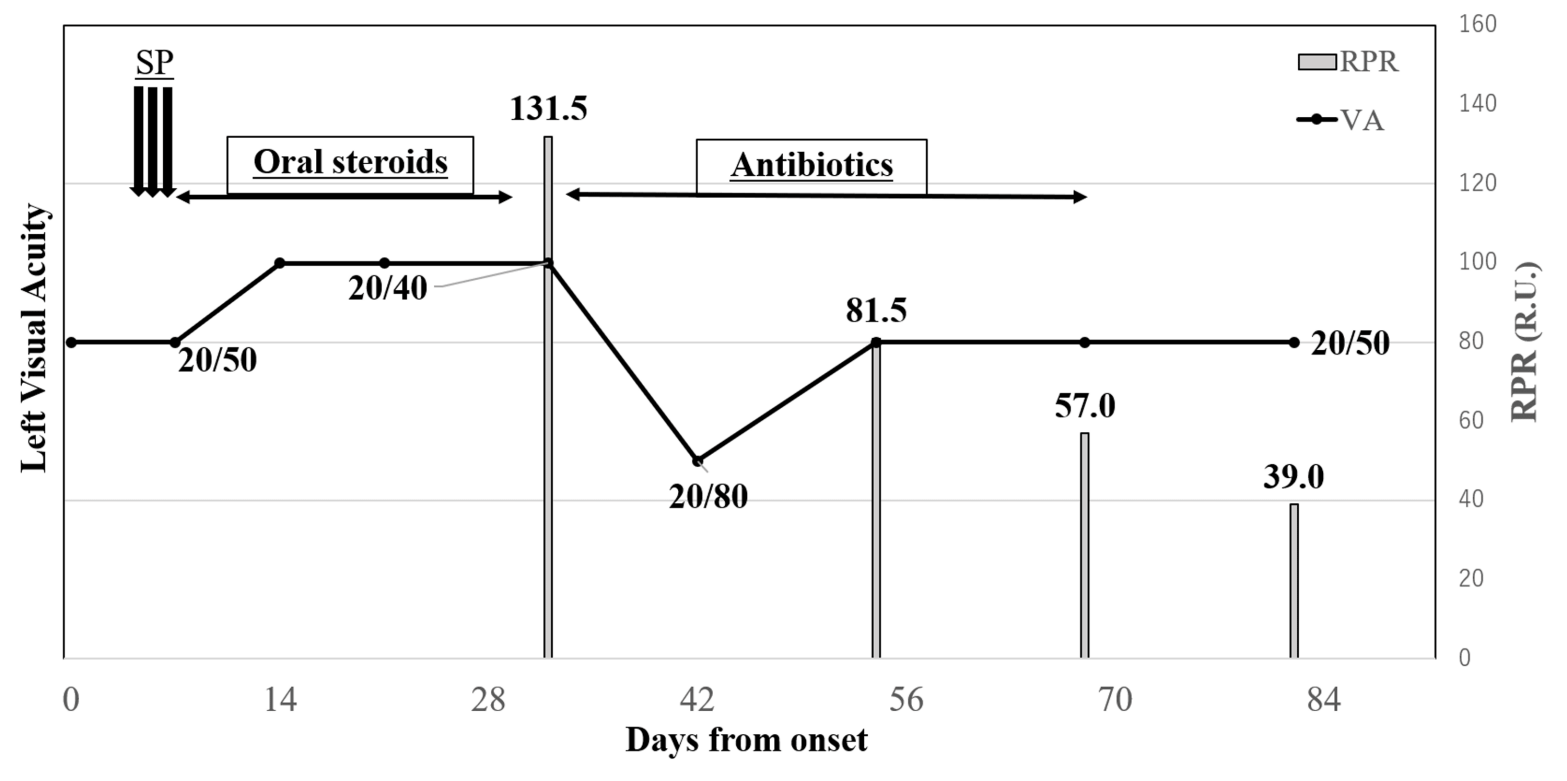
Changes in left visual acuity from onset and treatment details and changes over time from the start of RPR measurement. At the onset of optic papillitis, the left visual acuity was 20/50, with a central scotoma. After steroid pulse therapy was completed, the optic papillitis improved on OCT and MRI, and the left visual acuity improved to 20/40. After oral steroid therapy was discontinued, RPR was found to be high at 131.5 R.U., and antibiotics were initiated. After antibiotics were initiated, OCT showed the appearance of left outer retinopathy and the left visual acuity decreased to 20/80. After continuing antibiotics for five weeks, RPR decreased to 57.0 R.U., and the outer retinopathy improved on OCT, as did the central scotoma in the visual field, but the left visual acuity only improved to 20/50, the same as at the onset. SP: steroid pulse, RPR: rapid plasma regain, VA: visual acuity, OCT: optical coherence tomography, MRI: magnetic resonance imaging.

A few days after starting antibiotics, the left visual acuity had decreased from 20/40 to 20/80, and OCT findings showed disruption and loss of the macular EZ and inferior ELM (Figure 4A). Oral antibiotics were continued while steroids were discontinued, and subjective symptoms and the left visual acuity gradually improved. Approximately five weeks after the start of antibiotic treatment, the quantitative RPR, which reflects the disease activity of syphilis, was confirmed to have decreased to 57.0 R.U., less than half of the initial value, and antibiotic treatment was then discontinued (Figure 5). After antibiotics were discontinued, the left visual acuity had improved to 20/50, the central scotoma in GP that had been present before the start of treatment completely disappeared, the left CFF increased to 25 Hz, and the disruption and loss of EZ and ELM in the left eye's OCT showed a tendency to improve (Figure 4B). The timing of the decline in vision in the left eye and the appearance of the disruption and loss of EZ and ELM in OCT (Figure 4A), as well as the timing of improvement in vision and OCT findings (Figure 4B), were consistent, suggesting that vision correlated with the retinal changes seen on OCT and improved with antibiotic treatment.

The progress of the left visual acuity from the onset of left optic papillitis, the details of treatment, and the time-dependent change from the start of RPR measurement are shown in Figure 5. After antibiotic treatment was completed, the left visual field, MRI findings, and OCT findings all improved, while the final visual acuity was 20/50, the same as at the time of onset.

## Discussion

In this case, key clinical findings included unilateral optic disc swelling without signs of uveitis, a central scotoma, and a lack of response to initial steroid therapy. The diagnosis was complicated by the coexistence of diabetic retinopathy. These findings, combined with a positive syphilis serology, helped differentiate syphilitic optic papillitis from other causes of optic neuritis. Initially treated with steroid pulse therapy, the patient's optic disc swelling improved (Figures 2A, 2C), but her visual acuity worsened, and OCT showed disruption in the ellipsoid zone (Figure 4A). After starting antibiotic treatment with penicillin for syphilis, the outer retinal disruption began to improve (Figure 4B), and the patient’s visual acuity stabilized. These observations suggest that early antibiotic treatment is crucial in managing syphilitic optic papillitis, even in the presence of diabetic retinopathy. This case highlights the diagnostic challenge of ocular syphilis in the absence of typical uveitis. Clinicians should consider syphilis in the differential diagnosis of optic neuritis, especially when there is unilateral optic disc swelling and a lack of improvement with steroid therapy. Syphilis serology should be conducted early in such cases to avoid delayed diagnosis.

Ocular syphilis frequently presents with uveitis, although optic nerve lesions have also been reported [7,8,15,16]. Previous reports have shown that optic nerve lesions were in 78% of ocular syphilitic cases, and the most common was optic disc swelling [6], and that both optic papillitis and retrobulbar optic neuritis can occur in unilateral or bilateral eyes [8,15,16]. Optic neuritis associated with syphilis infection often presents as neurosyphilis, and as it progresses, optic nerve atrophy occurs, resulting in a poor prognosis for visual function [9]. Neurosyphilis is diagnosed based on abnormalities in cerebrospinal fluid (CSF) tests. In this case, since there were no obvious signs of neurological abnormalities other than visual impairment at the time of onset, neurosyphilis was not suspected and a CSF test was not performed. However, if neurosyphilis had been latent, administering steroid pulses prior to antibiotics could have worsened not only the visual impairment but also the overall condition, including the cranial nervous system. It was advisable to perform a CSF test before treatment when optic neuritis develops and syphilis is positive.

Regarding structural changes in the retina due to ocular syphilis, OCT findings of outer and inner retinal disorders have been reported [11,12]. While outer retinopathy often improves with appropriate antibiotic treatment, disruption or loss of EZ, seen in 61% of cases, if persistent, is associated with a poor visual prognosis [12]. ASPPC, which is a particular type of syphilis-related retinal disorder, typically presents as one or more placoid-shaped, pale yellowish subretinal lesions in the macula, and is characterized by hyper-autofluorescence due to RPE defect accompanied by accumulation of lipofuscin [17]. FA is characterized by early hypo-fluorescence mixed with late hyper-fluorescence, forming leopard spots [10]. The pathology of ASPPC is thought to involve infection of the RPE and choroid by *Treponema pallidum*, as well as inflammation and circulatory failure due to autoimmunity [14], but there are many unknowns. ASPPC was previously thought to be more likely to develop in immunosuppressed conditions such as those associated with HIV, but it has also been reported that it can develop in immunocompetent patients [8,13]. In this case, fundus examinations during the course of the disease and FA at the time of the onset of papillitis did not reveal any of the typical findings of ASPPC described above (Figures 1A, 1B). OCT findings of outer retinal disorder were not clear at the time of the onset of papillitis (Figures 2A, 2B), and so we considered the possibility that the outer retinal disorder that appeared after steroid administration was not due to diabetic retinopathy but was due to syphilis infection. Since outer retinal disorder may remain after the resolution of macular edema due to diabetic retinopathy, it became difficult to diagnose this as a new finding due to syphilis infection. Changes in OCT findings due to syphilis infection should be observed carefully, especially in cases with other retinal diseases.

The first choice of treatment for syphilis is a penicillin antibiotic, preferably taken orally for four weeks or, particularly in the case of neurosyphilis, intravenously for 10-14 days [18]. The timing of the end of antibiotic administration is determined by the decrease in the quantitative RPR value as an indicator of cure. Specifically, when the quantitative RPR value falls to half or less of the initial value, it is judged that the disease activity has decreased and treatment is effective. In this case, oral antibiotics were continued for five weeks. Antibiotics were discontinued after the RPR value fell to half or less of the initial value and subjective symptoms and OCT findings improved (Figure 4B). After antibiotic treatment, visual acuity improved to the same level as before the appearance of outer retinopathy, along with the improvement of OCT findings, and the central scotoma disappeared. It is possible that antibiotic treatment had a certain therapeutic effect on outer retinopathy caused by syphilis infection.

On the other hand, there is disagreement as to whether the use of steroids in combination is effective in treating ocular syphilis. In a case in which vision declined due to ASPPC after the onset of optic papillitis, visual function improved when steroids were added after the initiation of antibiotic treatment [8]. In contrast, in a case that developed with outer retinopathy and was initially thought to be AZOOR and was first treated with steroids, the patient was later found to be positive for syphilis, and visual function improved after the addition of antibiotics [13]. For both syphilis-related optic papillitis and ASPPC, the combination of antibiotics and steroids may be effective; however, caution is required as there has been a report of oral steroids inducing the onset of ASPPC [19]. This case was initially thought to be idiopathic optic neuritis, and steroid pulse therapy was administered first. The optic papillitis disappeared after steroid administration, but if syphilis infection had been latent from the time of the onset of papillitis, it may have worsened. In addition, since outer retinopathy appeared after the disappearance of papillitis, it was thought that its appearance may have been induced by the prior administration of steroids. When diagnosing optic neuritis, syphilis serology should be performed to differentiate for differentiation, and if the result is positive, antibiotics should be administered in preference to steroids.

This case had originally had a decline in visual acuity due to the effects of diabetic retinopathy, but no outer retinopathy was observed at the time of onset. Therefore, the initial decline in visual acuity and CFF and the central scotoma in the visual field were thought to be caused by unilateral optic papillitis. We believe that steroid treatment was somewhat effective in treating the deterioration of visual function due to optic papillitis, optic disc edema, and MRI findings. On the other hand, outer retinopathy, which was not initially observed, developed after steroid administration and was thought to have developed due to the presence of ocular syphilis, causing a further deterioration in visual acuity. OCT confirmed that the outer retinopathy had improved with additional antibiotic administration, and there was a certain improvement in visual function as well. There have been no reported cases of diabetic retinopathy and ocular syphilis occurring concomitantly, and this case was atypical in that the outer retinopathy appeared after optic papillitis. This case may suggest that determining whether outer retinopathy is originally present due to diabetic retinopathy using OCT is important in determining whether ocular syphilis has occurred concomitantly.

## Conclusions

We have experienced a case of ocular syphilis complicated by unilateral optic papillitis and outer retinopathy. This case highlights the diagnostic complexity of ocular syphilis, especially in patients with pre-existing retinal conditions like diabetic retinopathy. Clinicians should consider syphilis in the differential diagnosis of optic neuritis when traditional treatments, such as steroids, fail to improve symptoms. In this case, although treatment with penicillin antibiotics resulted in clinical improvement and stabilization of visual function, the patient’s final visual acuity did not fully return to baseline, underscoring the importance of early diagnosis and intervention. Diagnostic tools such as OCT, MRI, and syphilis serology were critical in both the initial diagnosis and in tracking the response to treatment. Regular monitoring of serological markers and imaging findings is essential for evaluating the success of therapy in such complex cases. This case illustrates the importance of ruling out infectious causes, such as syphilis, before initiating steroid therapy in patients with optic neuritis. Early antibiotic treatment is essential to prevent the worsening of syphilitic optic papillitis.
